# Deep brain stimulation in *VPS13C*-associated Parkinson’s disease: a longitudinal case study

**DOI:** 10.1016/j.prdoa.2026.100445

**Published:** 2026-04-21

**Authors:** Hiroyuki Sumikura, Naoki Tani, Hiroyo Yoshino, Nobutaka Hattori, Manabu Sakaguchi

**Affiliations:** a Department of Neurology, Osaka General Medical Center, 3-1-56 Bandai-higashi, Sumiyoshi-ku, Osaka city 558-8558, Osaka, Japan; b Department of Neurosurgery, The University of Osaka, 2-2 Yamadaoka, Suita 565-0871, Osaka, Japan; c Research Institute for Diseases of Old Age, Graduate School of Medicine, Juntendo University, 2-1-1 Hongo, Bunkyo-ku, Tokyo 113-8421, Japan; d Department of Neurology, Faculty of Medicine, Juntendo University, 3-1-3 Hongo, Bunkyo-ku, Tokyo 113-8431, Japan

**Keywords:** VPS13C, Early-onset parkinsonism, Deep brain stimulation, MIBG scintigraphy, Autonomic denervation, genetic Parkinson disease, Surgicogenomics

## Abstract

• VPS13C parkinsonism showed initial STN-DBS benefit then rapid decline.
• Motor, cognitive, and psychiatric deterioration occurred after DBS.
• Cardiac MIBG converted from normal uptake to denervation over 7 years.
• Findings support genetic testing before DBS in early-onset PD.
• Surgicogenomics may guide DBS candidate selection in genetic PD.

We report a female with early-onset parkinsonism owing to a homozygous *VPS13C* splice-site variant (c.7063-2A>G) who underwent bilateral subthalamic nucleus deep brain stimulation (STN-DBS) with initially excellent motor benefits, followed by rapid deterioration across motor, cognitive, psychiatric symptoms, as well as a unique longitudinal transition on ^123^I metaiodobenzylguanidine (MIBG) cardiac scintigraphy from normal to denervation within 7 years.

A right-handed female, born to consanguineous parents, the patient had normal early development. Family history was unremarkable, suggesting autosomal recessive inheritance. The patient’s symptoms began at age 24 with dystonic toe flexion and left predominant parkinsonism. At age 25, the patient was diagnosed with early-onset Parkinson’s disease (EOPD). The clinical course is detailed in **Supplementary Table S1**. Progressive wearing-off emerged by 28–29 years of age and became disabling despite optimized oral therapy and apomorphine rescue. At age 30 years, given robust levodopa responsiveness (Movement Disorder Society-Unified Parkinson’s Disease Rating Scale [MDS-UPDRS]-III improvement 82%, Hoehn & Yahr [H&Y] 2 and MDS-UPDRS-III 13 in “on” state vs. H&Y 5 and MDS-UPDRS-III 72 in “off” state), the patient underwent bilateral STN-DBS (Activa PC, Medtronic). Immediately after surgery, the wearing-off disappeared, and the patient resumed community activities and participated in vocational training for several years. At age 31 years, screening for rapid eye movement sleep behavior disorder (RBD) using the RBD Screening Questionnaire (RBDSQ) and Mayo Sleep Questionnaire was negative. Cognitive performance was largely preserved (Mini-Mental State Exam [MMSE] 30/30, Frontal Assessment Battery [FAB] 15/18, Addenbrooke’s Cognitive Examination-Revised [ACE-R] 85/100) with only mild executive dysfunction, which did not interfere with daily life. Genetic testing at 33 years of age identified a novel homozygous vacuolar protein sorting 13 homolog C (*VPS13C*) splice acceptor variant (c.7063-2A > G) (**Supplementary Material**). This variant was not registered in ClinVar database, and was classified as “Pathogenic” (PVS1, PM2, and PP3) according to the ACMG guideline, given its location at a canonical splice site, its absence in gnomAD and the Tohoku Medical Megabank Organization 60KJPN databases, and deleterious predictions by multiple in silico tools. Functional validation or segregation studies with family members have not been conducted. From age 35 to 36 years, the patient experienced abrupt clinical declines in motor, cognitive, and psychiatric status despite stable stimulation and medication, including worsening gait with Pisa tendency and frequent falls, dysarthria, apathy and anhedonia, and pronounced disinhibition with hypersexuality. Replacing the pulse generator with a sensing-enabled neurostimulator (Percept RC, Medtronic) yielded no benefit. At age 36 years, the patient was hospitalized after being found wandering in public wearing only underwear and taken into protective custody by police. Neuropsychological scores fell (MMSE 11/30, FAB 6/18, and ACE-R 48/100). Brain magnetic resonance imaging and computed tomography performed at age 25–36 years did not show progressive brain atrophy (Fig. 1). Strikingly, cardiac MIBG transitioned from normal (age 29; heart-to-mediastinum [H/M] early/delayed 3.22/3.35) to denervation (age 36; H/M 1.92/1.79) (Fig. 1), suggesting rapid postganglionic sympathetic denervation over only 7 years. The patient reached H&Y stage 4, necessitating discontinuation of vocational activities and the requirement for 24-h supervision.

**Fig. 1:**
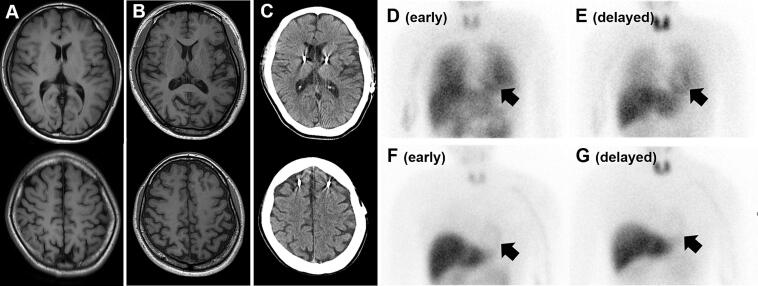
(A–C) Serial brain imaging showing no significant cerebral atrophy: T1-weighted MRI at 25 (A) and 31 (B) years of age, and CT at age 36 (C). (D–G) ^123^I-MIBG myocardial scintigraphy. Initial scans at age 29 (D, E) show normal cardiac uptake (heart-to-mediastinum [H/M] ratio: early, 3.22; delayed, 3.35). Follow-up scans at age 36 (F, G) reveal markedly decreased uptake (H/M ratio: early, 1.92; delayed, 1.79). MRI, magnetic resonance imaging; CT, computed tomography; H/M heart-to-mediastinum; MIBG, metaiodobenzylguanidine.

*VPS13C* loss–of–function is an established cause of autosomal–recessive EOPD with frequent cognitive and psychiatric features, broad α–synuclein pathology at autopsy, and occasional phenotypic overlap with dementia with Lewy bodies (DLB). Including this case, 28 patients with *VPS13C* have been reported. Among them, 21/28 (75%) and 7/28 (25%) showed Parkinson’s disease (PD) and DLB, respectively, with early cognitive impairment in 18/24 (75%) and psychiatric features in 11/19 (58%) of these patients. Our findings suggest that the durability of motor benefits from DBS may be limited in some *VPS13C*-PD cases due to the rapid subsequent deterioration across motor, cognitive, and psychiatric domains. This subacute decline despite optimized stimulation and medication indicates natural disease progression rather than a DBS-related effect. Among two previously reported patients with *VPS13C*-PD undergoing DBS [1], [2], one experienced significant benefit in the initial 2 years and the other a poor response; however, *peri*-operative details and longitudinal courses are limited. While DBS is a good option for early-onset and genetic PD, particularly Parkin RBR E3 ubiquitin protein ligase (*PRKN*)-PD and leucine-rich repeat kinase 2 (*LRRK2*)-PD, which often achieve favorable long-term outcomes, indications for GBA1 (*GBA*)-PD should be determined with greater caution, given the risk of rapid postoperative deterioration in motor, cognition, and QOL [3]. Regarding DBS surgical timing and prognostic factors, Wang et al. highlighted the risk of relying solely on levodopa responsiveness for candidate selection, specifically noting that short disease duration, rapid symptom progression, and higher baseline motor severity are associated with poorer outcomes [4]. Our patient’s clinical course aligns with these finding, suggesting that indications for *VPS13C*-PD may warrant even greater caution than those for *GBA*-PD. Although phenotypic heterogeneity is a critical consideration in genetic PD, this underscores the importance of preoperative genetic evaluation—“surgicogenomics”—in optimizing patient selection, prognosticating outcomes, and tailoring personalized medicine.

The two previously reported cases with available MIBG data showed reduced uptake [5], but no longitudinal conversions have been documented. The present case is consistent with brain-first PD based on the absence of RBD and preserved cardiac MIBG uptake 5 years after symptom onset. Although EOPD is frequently associated with a brain-first type, further investigation regarding peripheral organs and the autonomic nervous system is required to determine whether *VPS13C* (PARK23) follows a brain-first or body-first type. The delayed H/M ratio on MIBG decreased by 46% over 7 years in the present case, representing an annual decline of 6.5%. This progression is more rapid than the approximately 4% annual decline reported in sporadic PD [6]. Future histopathological investigations of peripheral organs in post-mortem *VPS13C*-PD patients are essential to clarify the α-synuclein pathology beyond the central nervous system.

This case highlights two practice points. First, the *VPS13C* genotype is a critical factor in prognosticating DBS outcomes. Despite excellent levodopa responsiveness and initial surgical success, clinicians must inform patients and families that medium-to-long-term outcomes are uncertain because of possible rapid motor, cognitive, and psychiatric decline. Second, longitudinal MIBG assessments suggest that peripheral autonomic integrity in the early stage of *VPS13C*-PD can be followed by rapid denervation.

## Ethics Statement

The study was conducted in accordance with the principles of the Declaration of Helsinki. Ethical approval was obtained from the Research Ethics Board of our hospital.

## 2 Patient Consent Statement

Written informed consent was obtained from the patient for publication.

## 3 Funding Source

This research did not receive any specific grant from funding agencies in the public, commercial, or not-for-profit sectors.

## 4 Data statement

All relevant data are provided in the present report.

## CRediT authorship contribution statement

**Hiroyuki Sumikura:** Writing – original draft, Visualization, Investigation, Data curation, Conceptualization. **Naoki Tani:** Writing – review & editing, Validation, Investigation, Data curation, Conceptualization. **Hiroyo Yoshino:** Writing – review & editing, Validation, Investigation, Data curation. **Nobutaka Hattori:** Writing – review & editing, Supervision. **Manabu Sakaguchi:** Writing – review & editing, Validation, Supervision.

## Declaration of competing interest

The authors declare that they have no known competing financial interests or personal relationships that could have appeared to influence the work reported in this paper.
