# Supplementary material for: Deep brain stimulation in VPS13C-associated Parkinson’s disease: a longitudinal case study
Source: Clin Park Relat Disord. 2026 Apr 21;14:100445. doi: 10.1016/j.prdoa.2026.100445 (PMC13126465; doi:10.1016/j.prdoa.2026.100445)
Supplement: Supplementary Data 1 [file mmc1.docx]

**Table S1. Longitudinal Clinical Timeline of the Patient in the Present Report**

| Adolescence | No developmental delays during infancy or early childhood were noted. The patient participated in a baton club in elementary school and a basketball club in junior high school. Academic performance was not a strength. After graduating from high school, the patient attended a clerical vocational school and subsequently worked as a clerk until 24 years of age. |
| --- | --- |
| Age 24 years | Initial symptoms were flexion of the left toes suggestive of dystonia and stiffness in the left upper and lower extremities. |
| Age 25 years | At the initial neurological evaluation, the patient presented with left-sided resting tremor, muscle rigidity, and bradykinesia. Initial dopamine transporter imaging (DaTscan; single-photon emission computed tomography with ioflupane [^123^I-FP-CIT SPECT]) revealed a right-dominant reduction in striatal uptake (specific binding ratio (SBR): right 4.38, left 6.72), suggesting presynaptic dopaminergic denervation. A diagnosis of early-onset Parkinson’s disease was made, and treatment with extended-release pramipexole was initiated. |
| Age 28 years | Wearing-off emerged. |
| Age 29 years | Wearing-off phenomenon exacerbated, characterized by frequent off periods with complete immobility that were relieved by rescue doses of levodopa. The patient exhibited an excellent clinical response to levodopa. Initial ^123^I-metaiodobenzylguanidine (MIBG) myocardial scintigraphy showed normal cardiac uptake (heart-to-mediastinum [H/M] ratio: early 3.22, delayed 3.35), suggesting preserved postganglionic sympathetic nerve terminals. The second DaTscan revealed decreased accumulation (SBR: right 2.42, left 4.25), indicating progression of presynaptic dopaminergic denervation over 4 years. |
| Age 30 years | Subcutaneous apomorphine (3 mg) was also highly effective for off periods involving complete immobility. Wearing-off management was challenging, requiring complex medication adjustments including levodopa (500 mg/day in seven divided doses), entacapone (300 mg in three divided doses), pramipexole (3.75 mg), rotigotine (9 mg), istradefylline (40 mg), and subcutaneous apomorphine (3 mg, 6–8 times daily). Although the patient was able to go outdoors during on periods, severe daytime off periods frequently rendered the patient bedbound and unable to handle medication packaging or leave the restroom. Additionally, nocturnal akinesia prevented the patient from turning over in bed. Levodopa-induced dyskinesia (LID) was mild and did not interfere with activities of daily living (ADL). The patient exhibited a strong craving for levodopa suggestive of dopamine dysregulation syndrome. The patient developed visual hallucinations, mistaking a hung coat for a person and speaking to it. Because of severe motor fluctuations refractory to optimized pharmacotherapy, the patient requested deep brain stimulation (DBS). Preoperative evaluation revealed severe wearing-off but remarkable levodopa responsiveness (82% improvement), in which the “off” state (Hoehn and Yahr [H&Y] stage 5, Movement Disorder Society-Unified Parkinson’s Disease Rating Scale [MDS-UPDRS]-III 72) significantly improved to the “on” state (H&Y stage 2, MDS-UPDRS-III 13) following levodopa administration, along with MDS-UPDRS Part I and II scores were 20 and 16, respectively. The patient underwent bilateral subthalamic nucleus DBS using an Activa PC (Medtronic, Minneapolis, MN, USA) with stimulation settings of 1.9 V, 60 μs, and 130 Hz for the right side, and 2.0 V, 60 μs, and 130 Hz for the left side. Postoperatively, motor complications, including wearing-off and LID, were resolved. |
| Age 31 years | At the time of the initial visit to our department, the patient’s antiparkinsonian medications had been reduced to levodopa (500 mg/day in seven divided doses), entacapone (300 mg), and pramipexole (0.375 mg). Subcutaneous apomorphine, rotigotine, and istradefylline had already been discontinued. Following DBS treatment, the patient no longer experienced wearing-off episodes, and an “on” state (H&Y stage 2) was consistently maintained throughout the day. Mild LID persisted as diphasic dyskinesia (beginning-of-dose LID). The patient remained independent in ADLs and independently used public transportation such as trains and buses. MDS-UPDRS Part I, II, and III scores were 12, 4, and 6, respectively. The patient was employed at a Type B support center for continuous employment, responsible for online sales for a clothing business. Rapid eye movement sleep behavior disorder (RBD) was ruled out, with a score of 2 on the RBD Screening Questionnaire (RBDSQ) and a “No” response to Question 1 of the Mayo Sleep Questionnaire (MSQ). Neuropsychological testing revealed 30/30 on the Mini-Mental State Examination (MMSE) and 15/18 on the Frontal Assessment Battery (FAB). The Addenbrooke’s Cognitive Examination-Revised (ACE-R) total score was 85/100 (attention/orientation 18, memory 16, fluency 11, language 26, and visuospatial 14). The patient exhibited a tendency to give up easily, frequently responding with “I don’t know” without sufficient consideration. These findings, along with impaired working memory and reduced verbal fluency, indicated mild frontal executive dysfunction. Psychiatric symptoms included levodopa craving and oral discomfort. Autonomic involvement was limited to constipation, with no urinary frequency or orthostatic hypotension (negative Schellong test). |
| Age 33 years | Genetic analysis identified a novel homozygous variant (c.7063-2A>G) in *VPS13C*. |
| Age 34 years | The patient exhibited no wearing-off, while non-troublesome beginning-of-dose LID remained present. The motor condition remained stable in an “on” state (H&Y stage 2), allowing for regular activities such as cycling to a fitness gym. The medications included levodopa (400 mg/day in eight divided doses) and opicapone. The DBS settings remained stable, with stimulation parameters of 2.7 V, 60 μs, and 130 Hz on the right side, and 2.1 V, 60 μs, and 130 Hz on the left side. |
| Age 35 years | A subacute decline in motor, cognitive, and psychiatric status was observed. The patient developed worsening small-step gait, postural instability including Pisa syndrome (rightward leaning) and frequent falls, apathy, and anhedonia, characterized by reduced spontaneous activity and loss of interest in hobbies, such as watching favorite animations. The disease severity progressed to H&Y stage 3. Following dysarthria emergence, the stimulation site of the DBS shifted dorsally, and the stimulation intensity was reduced; however, these adjustments were ineffective. The implantable pulse generator was replaced with the Percept RC (Medtronic), a sensing-enabled neurostimulator, which did not significantly alter clinical motor, cognitive, or psychiatric symptoms. Cognitive decline became prominent. The patient exhibited speech with unclear intent. Dressing apraxia developed. The patient became unable to distinguish between numbers and alphabets on a keyboard, and work performance at the disability support center deteriorated due to frequent errors. The Beck Depression Inventory-II (BDI-II) was discontinued due to a lack of patient cooperation. |
| Age 36 years | The patient was found wandering in public in a state of undress (wearing only underwear) and was detained by the police for safety and hospitalized at our institution. Motor function declined to H&Y stage 4 with a markedly unstable gait, including toe walking, instability, and repeated in-hospital falls. MDS-UPDRS scores for Part I, II, and III showed marked worsening, reaching 34, 32, and 59, respectively. The second ^123^I-MIBG myocardial scintigraphy showed decreased tracer uptake (H/M ratio: early 1.92, delayed 1.79), indicating that the postganglionic cardiac sympathetic nerve terminals had converted from normal to denervated over 7 years. Autonomic symptoms included constipation and urinary frequency due to overactive bladder; however, the Schellong test was negative and no orthostatic hypotension was observed. Neuropsychological testing showed severe cognitive decline: MMSE 11/30, FAB 6/18, and ACE-R 48/100 (attention/orientation 12, memory 6, fluency 2, language 22, and visuospatial 6). The patient exhibited prominent neuropsychiatric symptoms, including impulse control disorder manifesting as sexual disinhibition, characterized by sexually suggestive remarks and inappropriate physical contact (e.g., touching or hugging) toward male healthcare staff. Additionally, aimless wandering in the ward corridors was observed; when prompted, the patient could not state a destination. Episodes of psychomotor retardation were also noted, during which the patient would remain stationary for several minutes. When engaged in coloring, the patient used only a single color and performed the task in a disorganized manner, suggesting constructional apraxia, visuospatial dysfunction, and executive dysfunction. Furthermore, impaired persistence and apathy were evident, as the patient ceased the coloring task despite it being incomplete. When participating in card games, the patient could not retain the rules and required frequent external prompting and repetitive instruction for every turn, suggesting executive dysfunction, working memory deficit, and attentional deficit. The patient’s psychiatric condition stabilized with quetiapine (37.5 mg/day), allowing for home discharge. Motor function remained H&Y stage 4. Orthostatic hypotension remained absent (negative Schellong test). |

**Supplementary Material**

**Genetic Analysis**

Next-generation sequencing was used to analyze the coding regions and splice sites of Parkinson’s disease-associated genes (including *SNCA*, *PRKN*, *UCH-L1*, *PINK1*, *DJ-1*, *LRRK2*, *ATP13A2*, *GIGYF2*, *HTRA2*, *PLA2G6*, *FBXO7*, *VPS35*, *EIF4G1*, *DNAJC6*, *SYNJ1*, *DNAJC13*, *CHCHD2*, *VPS13C*, *GCH1*, *NR4A2*, *RAB7L1*, *BST1*, *C19orf2*, *RAB39B*, *VPS13A*, *VPS13B*, *VPS13D*, *LRP10*, *ELOVL7*, *PSAP*, and *UQCRC1*) and dementia-related genes (including *MAPT*, *PSEN1*, *GRN*, *APP*, and *APOE*).

Very rare variants (minor allele frequencies <0.5%) were identified using gnomAD and the Tohoku Medical Megabank Organization 60KJPN as reference panels for the Japanese population.

We prioritized variants located in coding regions or within 10 bp of splice sites.

Deleterious variants were defined as a Combined Annotation-Dependent Depletion score of >15 and a “damaging” prediction by at least two in-silico tools (AlphaMissense, MutationTaster 2021, SIFT, PolyPhen-2, and SpliceAI).

A novel homozygous canonical splice site variant in *VPS13C* (c.7063-2A>G) was identified, which was predicted to cause aberrant splicing such as exon skipping and was also suspected to be involved in disease pathogenesis. Based on the ACMG criteria, the variant was classified as Pathogenic: PVS1; Null variant at a canonical splice site in a gene where loss of function is a known mechanism. PM2; Absent from gnomAD and the 60KJPN Japanese reference panel. PP3; Consistent deleterious predictions by multiple in silico tools.
